# On the Aggregation and Nucleation Mechanism of the Monoclonal Antibody Anti-CD20 Near Liquid-Liquid Phase Separation (LLPS)

**DOI:** 10.1038/s41598-020-65776-6

**Published:** 2020-06-01

**Authors:** Elvira Pantuso, Teresa F. Mastropietro, Maria L. Briuglia, Charline J. J. Gerard, Efrem Curcio, Joop H. ter Horst, Fiore P. Nicoletta, Gianluca Di Profio

**Affiliations:** 1 National Research Council of Italy (CNR) - Institute on Membrane Technology (ITM), Via P. Bucci Cubo 17/C, 87036 Rende, CS Italy; 2 Department of Pharmacy, Health and Nutritional Sciences, University of Calabria, Via P. Bucci Edificio Polifunzionale, 87036 Rende, CS Italy; 3 EPSRC Centre for Innovative Manufacturing in Continuous Manufacturing and Crystallisation (CMAC), Strathclyde Institute of Pharmacy and Biomedical Sciences, Technology and Innovation Centre, University of Strathclyde, 99 George Street, Glasgow, G1 1RD UK; 4 Department of Environmental Engineering (DIAm), University of Calabria, Via P. Bucci Cubo 45/A, 87036 Rende, CS Italy; 5 Seligenda Membrane Technologies S.r.l., Via P. Bucci Cubo 45/A, 87036 Rende, CS Italy

**Keywords:** Protein aggregation, Biophysical chemistry, Crystal engineering, Protein-protein interaction networks, Surfaces, interfaces and thin films

## Abstract

The crystallization of Anti-CD20, a full-length monoclonal antibody, has been studied in the PEG400/Na_2_SO_4_/Water system near Liquid-Liquid Phase Separation (LLPS) conditions by both sitting-drop vapour diffusion and batch methods. In order to understand the Anti-CD20 crystallization propensity in the solvent system of different compositions, we investigated some measurable parameters, normally used to assess protein conformational and colloidal stability in solution, with the aim to understand the aggregation mechanism of this complex biomacromolecule. We propose that under crystallization conditions a minor population of specifically aggregated protein molecules are present. While this minor species hardly contributes to the measured average solution behaviour, it induces and promotes crystal formation. The existence of this minor species is the result of the LLPS occurring concomitantly under crystallization conditions.

## Introduction

A thorough understanding of protein aggregation behaviour in solution is crucial for the controlled manufacture and formulation of therapeutics and for the development of crystallization-based purification strategies for biological drugs^1–4^. Unfortunately, while the state diagram of protein-solvent systems can provide indications about the phase behaviour of biomacromolecules and their stability in solution, the intimate mechanism underlying protein nucleation and crystal growth have not been fully understood yet and, in many cases, it remains elusive. Protein aggregation is directly related to protein-protein interactions and may be strongly related to protein crystallization behaviour^5^. Since different types of intermolecular forces are involved in protein interactions, such as electrostatic, van der Waals and hydrophobic^6^, environmental factors like pH, ionic strength and the presence of additives, can drastically alter the intermolecular interaction profile of proteins in solution^7,8^.

Colloidal and conformational stabilities are the two main factors that govern the existence of a protein aggregate in a solution^9,10^. Colloidal stability is a delicate balance between repulsive and attractive forces between proteins, while conformational stability is related to the free energy difference between two molecular states, folded and partially or totally unfolded. Several investigations have found that the melting temperature (*T*_*m*_) and the osmotic second virial coefficient (*B*_22_) can respectively account for the protein conformational^11^ and colloidal^12^ stability. Both *T*_*m*_ and *B*_22_ can be experimentally determined by using correspondingly Dynamic Light Scattering (DLS) and Static Light Scattering (SLS) methods^13^. Aggregation induced by thermal unfolding can be monitored by measuring the average hydrodynamic diameter value as a function of temperature, while *B*_22_ describes the first deviation from ideal behaviour of dilute colloidal solutions^14^. A positive or negative value of *B*_22_ accounts for a net repulsive or attractive interaction balance, respectively, and could therefore be used to predict stable, crystallizing or precipitating protein solution conditions. According to the experimental crystallization slot of George and Wilson^15^, a protein solution would be: stable if *B*_22_ > −0.8 × 10^−4^ mL mol g^−2^; crystallizing if −8.4 × 10^−4^ < *B*_22_ < −0.8 × 10^−4^ mL mol g^−2^; precipitating if *B*_22_ < −8.4 × 10^−4^ mL mol g^−2^. Haas and Drenth^16^ postulated a modified crystallization slot, ranging from −0.9 × 10^−4^ to −0.35 × 10^−4^ mol mL g^−2^ for protein with a molecular size as high as 140 kDa.

The Z-potential is another indicator of colloidal stability, which measures the magnitude of the electrostatic or charge repulsion/attraction between particles^17^. It is related to the surface charge of the molecules, the adsorbed layer at the interface and the nature and composition of the surrounding environment. A large Z-potential value (|Z-potential| >30 mV) can be considered an indicator of colloidal stability. Recently, the use of the interaction parameter *k*_d_ has been also suggested as a more high-throughput means to quantify protein-protein interactions^18,19^.

In this work, we focus on the relationships between the crystallization propensity of Anti-CD20 in the Na_2_SO_4_/PEG400 solvent system of different compositions and its relation with some measurable parameters normally used to assess protein conformational and colloidal stability in solution, with the aim to understand the aggregation mechanism of this complex biomacromolecule. Anti-CD20 is a full-length monoclonal antibody (mAb) and it is a therapeutic protein extensively used for treatment of chronic lymphocytic leukaemia and non-Hodgkin’s lymphoma, which is commercialized with the brand name of Rituxan or Mabthera. Here, the crystallization of this biomolecule has been observed in a PEG400/Na_2_SO_4_/Water system near Liquid-Liquid Phase Separation (LLPS) conditions by both vapour diffusion and batch methods. LLPS is a very common phenomena for monoclonal antibodies^20^ and is of great interest because the factors which induce the separation of protein solutions into coexisting protein-poor and protein-rich phases are observed to play a central role in protein interaction and crystallization^21^. For several protein/PEG or protein/salt systems both the LLPS and the aggregation propensity of proteins in solution have been shown to coincide with net attractive interactions between molecules^22–24^. Monte Carlo simulations performed on systems that undergo phase separation demonstrated that the interactions between the proteins fall into the short-range regime and are strongly anisotropic^25^. Over time, the anisotropic interactions may be responsible for the evolution of the system from the phase separation to reversible aggregation conditions. In this situation, the LLPS has been speculated to behave as a metastable state towards nucleation^26–28^. In fact, the pathway for the formation of crystal nuclei drastically changes near the metastable low density–high density liquid region coexistence, with the free-energy barrier for crystal nucleation being strongly reduced. In this scenario, the crystal nucleation rate increases by many orders of magnitude over that predicted from classical nucleation theory^28^, thus generating a fast and diffuse crystallization that often results in poor-quality crystals.

The results of this investigation allowed us to gain further insights into the mechanism governing the nucleation-crystallization of a complex system such as the Anti-CD20 mAb.

## Methods

### Anti-CD20 crystallization experiments

Anti-CD20 monoclonal antibody (*M*_W_ = 144.5 kDa) was kindly provided by FUJIFILM Diosynth Biotechnologies (Billingham, UK) at high level of purity (>98% HPLC, >95% SDS-PAGE) in 0.025 M sodium citrate buffer at pH 6.5 and 0.154 M sodium chloride solution. Sodium sulphate anhydrous (purity ≥ 99.99%), PEG400, HEPES (purity ≥ 99.5%), TRIS (purity ≥ 99.5%), magnesium chloride hexahydrate (≥99%) and NaOH (purity ≥ 98%), from Sigma-Aldrich (Italy), were used without further purification. MilliQ water was used to prepare all solutions. Anti-CD20 was thawed for 2–3 hours in an 8 °C water bath before buffer exchange with HEPES 0.1 M and concentration by Amicon Ultra centrifugal filter tube (Ultra-4, cut-off 10 kDa, from Merck) at 6,000 g and 8 °C. Finally, the protein solution was diluted to the desired concentration by adding HEPES buffer 0.1 M. NaOH 1 M was used to adjust the pH to the desired value. The Anti-CD20 concentration was determined by measuring protein absorbance at 280 nm by UV-Vis spectrophotometer (extinction coefficient at 280 nm 237,380 M^−1^ cm^−1^).

Protocols suitable for crystallizing Anti-CD20 reproducibly by vapour diffusion and batch methods were developed in previous works^29,30^. Precipitant solutions were made by dissolving sodium sulphate in the HEPES buffer solution containing PEG400 at the proper concentration. All solutions were filtered through a 0.22 µm Anotop 10 Filter Unit (Whatman). In sitting-drop vapour diffusion crystallization experiments, drops made of 10 μL of precipitant solution and 10 μL of protein solutions were equilibrated in a sealed well against 1 mL of a hypertonic solution of MgCl_2_·6H_2_O 30 wt.% used as reservoir. For the batch crystallization experiments, a volume of protein solutions having a certain initial concentration was mixed with the required volume of precipitant solution to obtain a supersaturated solution of known composition (final volume 1 mL). All crystallization experiments were performed in a vibration-free refrigerator at 20 °C.

### Analysis of Anti-CD20 crystals

Crystals obtained from crystallization experiments were observed using an optical microscope (Eclipse LV 100ND, Nikon Instruments, Italy) equipped with a video camera, separated from the mother liquor by using a centrifuge (MiniSpin Plus Eppendorf model IVD) and centrifuge tubes (0.22 µm pore size) and extensively washed. Washing solution was made by 0.1 M HEPES, Na_2_SO_4_ and PEG400 whose concentration was optimized to avoid crystal solubilisation according to standard procedures. Washed crystals were dissolved by using 0.01 M TRIS-HCl, NaCl 0.15 M, pH 8.0 and analysed by UV spectrophotometry (Lambda EZ201, Perkin Elmer) to estimate protein concentration.

### Circular dichroism (CD) measurements

The far-UV CD spectra of Anti-CD20 under initial conditions, after buffer exchange with HEPES 0.1 M, and after mixing with the precipitant solution, were recorded between 200 and 300 nm on a Chirascan CD spectrometer (Applied Photophysics, UK) at 20 °C with a 1 nm bandwidth resolution and current time-per-point of 3 s. 100 μL of mAbs solution were added in a 0.1 mm path-length quartz cuvette that was placed in the spectrophotometer. Typically, three scans were recorded and baseline spectra were subtracted from each spectrum. Data were processed using Applied Photophysics Chirascan Viewer.

### Melting point and Z-potential assessment by DLS

Thermal stability (melting temperature, *T*_*m*_) of protein solutions was assessed by a Zetasizer Nano ZS instrument (Malvern) equipped with a 4 mW He–Ne laser at 632.8 nm and a detector placed at 173° in agreement to the proprietary NIBS (Non Invasive Back Scatter) technology. Samples were placed in quartz cuvettes (1 cm path-length), heated by a Peltier module and the average hydrodynamic diameter was measured as a function of temperature from 20 °C to 90 °C (temperature increment 0.5 °C). DLS can monitor the thermal denaturation of proteins, which leads to irreversible loss of their structure and function as the protein unfolds. When the average protein size is observed by DLS versus temperature, one will observe a significant increase in the average protein size for temperatures larger than the aggregation point, which is generally assumed as the onset of protein melting point.

For the Z-potential evaluations, the appropriate ratios of protein and buffer solutions were placed in disposable folded capillary cells (DTS 1070, Malvern), and then inserted into the Zetasizer instrument. The Z-potential was measured by laser Doppler micro-electrophoresis. Briefly, an electric field was applied to the protein solution and consequently each molecule moved with a velocity related to its Z-potential. The velocity was measured using a laser interferometric technique (M3-Phase analysis light scattering), which allowed the calculation of the electrophoretic mobility, and from this, the average Z-potential and Z-potential distribution.

### Light scattering measurements

Static and dynamic light scattering measurements were performed on protein solution samples with a concentration between 0.1 and 5.0 mg mL^−1^ in different crystallization cocktails. The Anti-CD20 concentration in these solutions was checked by measurement of the absorbance at 280 nm. All the solutions were filtered on a 0.22 µm filter (Anotop 10, Whatman) before measurement. The samples were placed in quartz ultra-low volume cuvettes (ZEN 2112, optical path 3 mm, Malvern) to measure with the Zetasizer Nano ZS instrument (Malvern) in static mode for the second virial coefficient *B*_22_ or dynamic mode for the protein diffusion coefficient *D*_m_. The light intensity and its time autocorrelation function were measured at 173° scattering angle. All measurements were performed at 20 °C after 2 min of equilibration using automatic time settings.

The Debye plots were generated by using Debye’s light scattering equation:

1 $$\frac{KC}{{R}_{\vartheta }}=\frac{1}{{M}_{W}}+2{B}_{22}C$$

where *R*_θ_ is the excess Rayleigh ratio of the protein in a solution with a protein concentration *C* and *M*_W_ is the average molecular weight of the protein. *K* is the optical constant and is defined as:

2 $$K=\frac{4{\pi }^{2}{n}^{2}{\left(\frac{dn}{dc}\right)}^{2}}{{N}_{A}{\lambda }^{4}}$$

where *n* is the solvent refractive index, d*n*/d*c* is the refractive index increment, *λ* is the wavelength of the incident light, and *N*_A_ is Avogadro’s number. The second virial coefficient *B*_22_ [mL mol g^−2^] was obtained from the slope of the linear Debye plot of *KC*/*R*_θ_ versus protein concentration *C*.

The static light scattering results for *B*_22_ were accepted if the following criteria were satisfied:

1. Signal to noise ratio >130%, where noise is solvent kcounts;
2. Increasing trend for signal kcounts vs. protein concentration.

The interaction parameter *k*_d_ [mL mg^−1^] and the self-diffusion coefficient *D*_*s*_ [m^2^ s^−1^] were determined by using linear fitting of the protein diffusion coefficient *D*_m_, determined from the dynamic light scattering measurements, plotted against the protein concentration *C* [mg mL^−1^], using the following Eq. 3^31^:

3 $${D}_{m}={D}_{s}(1+{k}_{d}C)$$

The determined interaction parameter *k*_d_ can be interpreted as the difference between a thermodynamic term related to the product 2*B*_22_·*M*_*W*_ and a hydrodynamic term (ξ_1_ + ν):

4 $${k}_{d}=2{B}_{22}{M}_{W}-({\xi }_{1}+\upsilon )$$

where *ξ*_1_ is obtained from the virial expansion of the concentration-dependent frictional coefficient and *ν* is the solvent viscosity.

Solutions that showed turbidity, indicating phase separation and crystallization/precipitation, were not used for measurements.

### Protein partitioning measurements

Anti-CD20 repartition between aqueous and PEG phases has been estimated by the method described by Kress *et al*.^32^. A solution (5 mL) containing Na_2_SO_4_ 1.1 M (13.54 wt.%) and PEG400 10.46% V/V (10.22 wt.%) in HEPES 0.1 M at pH 7.4, was added with 200 μL Anti-CD20 solution 10.3 mg mL^−1^ in the same buffer. After vigorous stirring for 5 minutes by vortex, the mixture has been left to rest overnight. The concentration of the protein in the two separated phases visually detected and in presence of excess of precipitated protein, was then assessed by UV spectrophotometry at 280 nm.

## Results and discussion

### Anti-CD20 crystallization and crystals analysis

**Figure 1:**
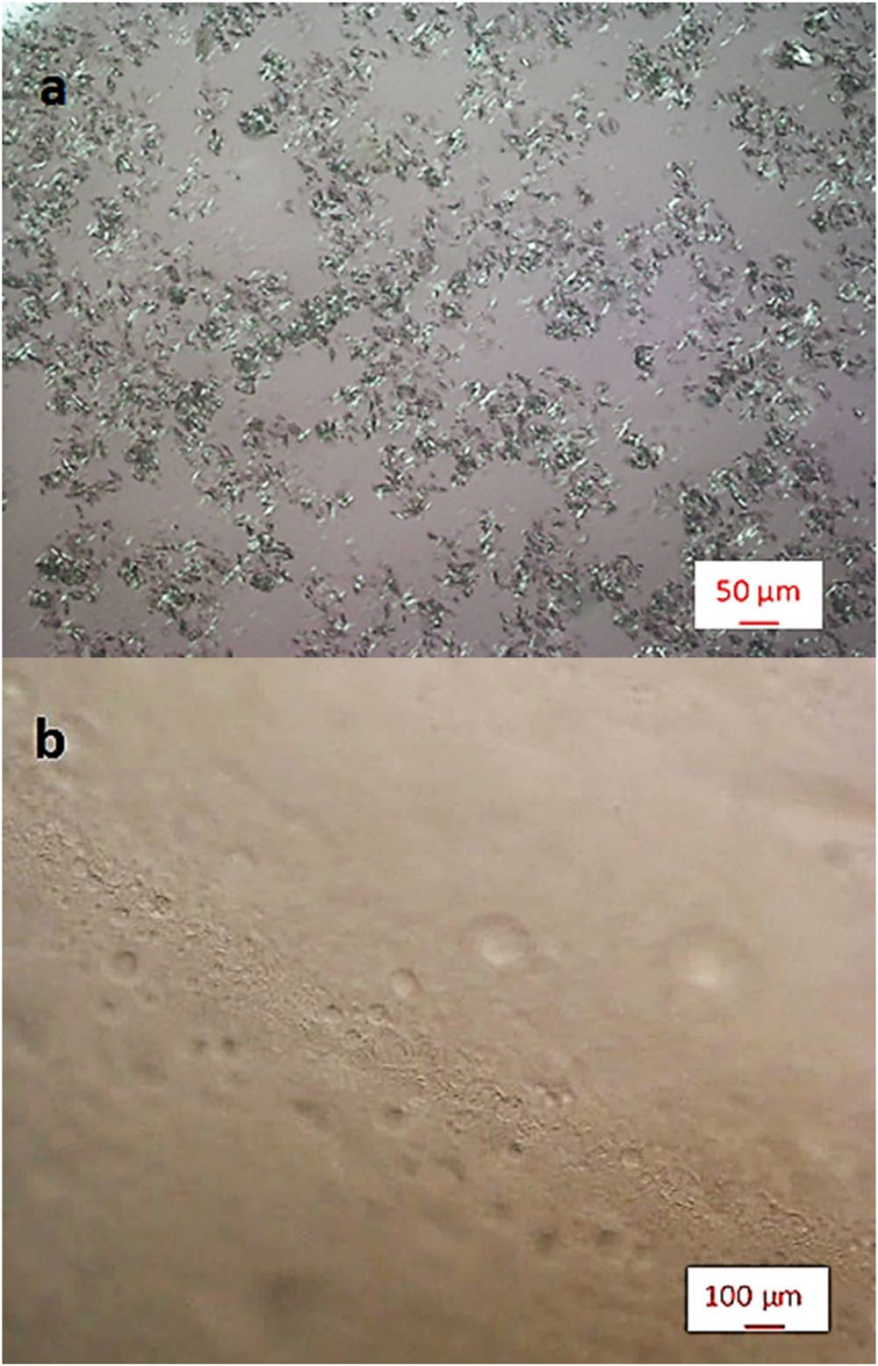
Optical microscope image of (**a**) crystalline Anti-CD20 monoclonal antibody obtained by sitting-drop vapour diffusion method and (**b**) Liquid-Liquid Phase Separation occurring just prior to crystallization. Solution composition near the crystallization point: 20 mg mL^−1^ Anti-CD20, 9.6% V/V PEG400, 0.86 M Na_2_SO_4_ in 0.1 M HEPES buffer at pH 7.4.

**Figure 2:**
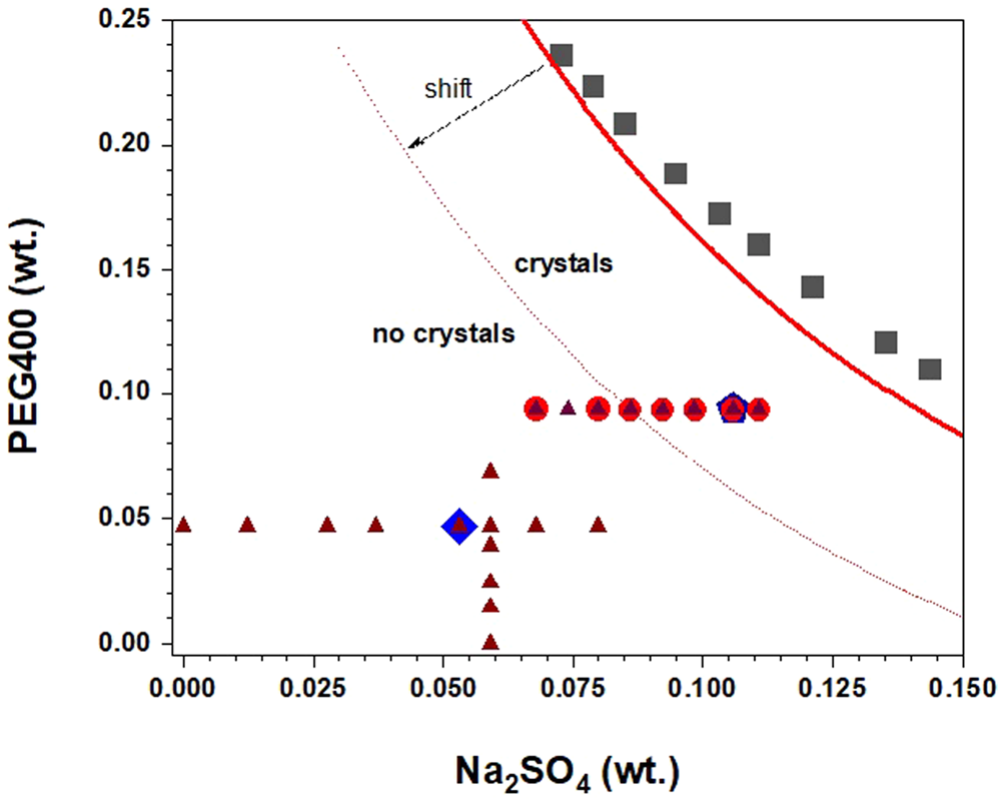
Experimental state diagram for the system PEG400/Na_2_SO_4_/H_2_O at 293.15 K. Squares: experimental liquid-liquid equilibrium data from ref. ^36^; solid line: asymptotic fitting to tie-lines data from ref. ^36^; circles: batch crystallization conditions reported in Table 1; diamond: starting solution composition in vapour diffusion tests; pentagon: ending solution composition in vapour diffusion tests; triangles: measurements points for *B*_22_; dotted line: shifted binodal curve in the presence of Anti-CD20 protein. Solution composition is expressed as weight fraction (wt.).

Anti-CD20 needle-like crystals (Fig. 1a) appear by sitting-drop vapour diffusion crystallization experiments using a 1:1 volume ratio mixture of a 20 mg mL^−1^ protein solution in HEPES buffer 0.1 M at pH 7.4 and a precipitant solution containing PEG400 9.6% V/V (9.4 wt.%) and Na_2_SO_4_ 0.86 M (10.6 wt.%) in the same buffer. Therefore, the initial composition of the drop in the vapour diffusion tests corresponds to 10 mg mL^−1^ Anti-CD20, 4.8% V/V PEG400, 0.43 M Na_2_SO_4_ in 0.1 M HEPES at a pH of 7.4 and is shown by the diamond symbol in Fig. 2. The crystals appear when the volume of the drop is nearly halved with respect to its initial volume. Accordingly, the final concentration of the components in the crystallizing drops is almost doubled that compared to the initial compositions and it is indicated by the pentagon symbol of Fig. 2.

Batch experiments in similar solution composition as that for crystallizing drops in the vapour diffusion tests (the term “similar” is used since the exact composition of the droplet at the crystallization point under equilibration with the reservoir solution it is not known), give also rise to crystals. Therefore, the batch working point coincides with the final conditions of the vapour diffusion tests. Since the batch composition does not vary with time, due to the absence of solvent evaporation, it is evident that the solution is supersaturated immediately upon mixing the protein and precipitant solutions, which results in a shorter crystallization time (12–18 h) than that observed with the sitting drop set up (36–48 h).

The UV-Vis and DLS measurements of washed and dissolved crystals confirmed their protein nature. All the analysed samples showed an intense band centred at 280 nm, typical of proteins absorption. The size distribution analysis by intensities show a single peak near 12 nm, size expected for Anti-CD20 protein molecules^33^, and a polidispersity <20%, indicating a high monodispersity.

Liquid-Liquid Phase Separation (LLPS) is observed nearly simultaneously with, or just prior to, crystals appearance (Fig. 1b) both in the vapour diffusion and batch crystallization trials. In accordance with other mAbs crystallization studies reported in literature^20,34,35^, also here it appears that the LLPS is a key step in Anti-CD20 crystallization.

Crystallization trials, aiming to investigate the effect of precipitant solution composition on crystal quality have been also performed by changing the starting amount of PEG400 and/or Na_2_SO_4_. As expected, the shape, size and density of crystals (number of crystals per unity of volume) are found to depend on buffer composition, initial protein concentration and crystallization method (sitting drop or batch). All these parameters have also shown to impact on the time required to observe crystallization. Table 1 shows the effect of the salt concentration on the crystal appearance for the batch experiments. Needles with a size of nearly 20 µm are the largest crystals obtained for starting solutions with 9.6% V/V PEG400 and 0.70–0.80 M Na_2_SO_4_. A further increase in Na_2_SO_4_ concentration produces a faster and massive protein nucleation in less than 24 hours and crystals of smaller size (<10 µm) with a spherulite-like morphology appear. For a Na_2_SO_4_ concentration of 0.9 M or larger, upon mixing the solution immediately turns opalescent due to a macroscopic LLPS in the solution, followed by a rapid protein precipitation as gel beads. On the opposite side, for the lowest amount of salt (Na_2_SO_4_ ≤ 0.65 M), the solutions remain clear for months.

**Table 1:** Mixed solution compositions, crystallization behaviour and crystal appearance in the batch crystallization experiments using an Anti-CD20 concentration 20 mg mL^−1^, 9.6% V/V PEG400 and 100 mM HEPES buffer at a pH of 7.4.

Na_2_SO_4_ [M]	Crystallization behaviour	Size [µm]	Shape
0.90	Opalescent solution, Instantaneous Precipitation	<1	gel beads
0.86	Precipitation/crystallization in 12–24 hours	<10	spherulite
0.80	Crystallization in 18–36 hours	<20	needle
0.75	Crystallization in 24–48 hours	20	needle
0.70	Crystallization in 24–48 hours	20	needle
0.65	Clear and stable solution	—	—
0.55	Clear and stable solution	—	—

Figure 2 shows the experimental state diagram for the system PEG400/Na_2_SO_4_/H_2_O at 20 °C indicating the loci of the points related to solution compositions used for batch crystallization tests (circles) of Table 1, together with the starting (diamond) and ending (pentagon) solution compositions in vapour diffusion tests. The figure also displays the experimental liquid-liquid equilibrium data and the asymptotic fitting to tie-lines data reported in ref. ^36^.

### SLS/DLS analysis

The protein stability in several formulation buffers but without precipitating agents (PEG and/or Na_2_SO_4_), was assessed by DLS, resulting in values for the melting point and Z-potential. As reported in Table 2, all samples are characterized by similar values of melting point, Z-potential, intensity weighted mean hydrodynamic size *Z*_*avg*_ and polydispersion index (PDI). All protein samples show low positive values of the Z-potential, indicating a reduced colloidal stability in the various buffer solutions. Nevertheless, due to a PDI close to 0.1, all samples can be considered monodisperse. No significant variations were observed in the sample aggregation state over time in the range 12–24 h. Accordingly, further SLS/DLS studies were performed by using HEPES as formulation buffer since it was used in previously published crystallization protocols^30^.

**Table 2:** Results for melting point (*T*_*m*_), Z-potential, intensity weighted mean hydrodynamic size (*Z*_avg_), and polydispersion index (*PDI*) from formulation buffers screening (Anti-CD20 concentration 20 mg mL^−1^; ^a^HEPES buffer 0.1 M, pH 7.4; ^b^Sodium Citrate buffer 0.035 M, NaCl 0.15 M, pH 6.5; ^c^TRIS buffer 0.1 M, NaCl 0.15 M, pH 8.0).

Buffer	T_m_ [°C]	Z-potential [mV]	*Z*_avg_ [nm]	*PDI*
HEPES^a^	68.5 ± 0.5	2.2 ± 0.7	11.3 ± 0.1	0.10 ± 0.03
Sodium Citrate^b^	68.5 ± 0.5	1.1 ± 0.2	12.2 ± 0.2	0.04 ± 0.03
TRIS^c^	69.0 ± 0.5	7.0 ± 1.0	12.63 ± 0.03	0.12 ± 0.01
Sodium Citrate/HEPES (1:1 Vol)	69.0 ± 0.5	0.19 ± 0.04	13.6 ± 0.2	0.15 ± 0.03
TRIS/HEPES (1:1 Vol)	69.0 ± 0.5	2.3 ± 1.0	12.13 ± 0.03	0.06 ± 0.01

To study the aggregation propensity on Anti-CD20 in HEPES buffer and in various PEG400/Na_2_SO_4_ solution compositions, SLS and DLS measurements were performed on freshly prepared samples allowing to determine the second virial coefficient *B*_22_ and the interaction parameter *k*_d_, respectively. The value of *KC*/*R*_θ_ was determined as a function of protein concentration, ranging from 0.1 to 5.0 mg mL^−1^, by SLS. Results were used to evidence the combined effect of PEG400 and Na_2_SO_4_ on Anti-CD20 colloidal stability and to compare these results with effective crystallization outcomes.

**Figure 3:**
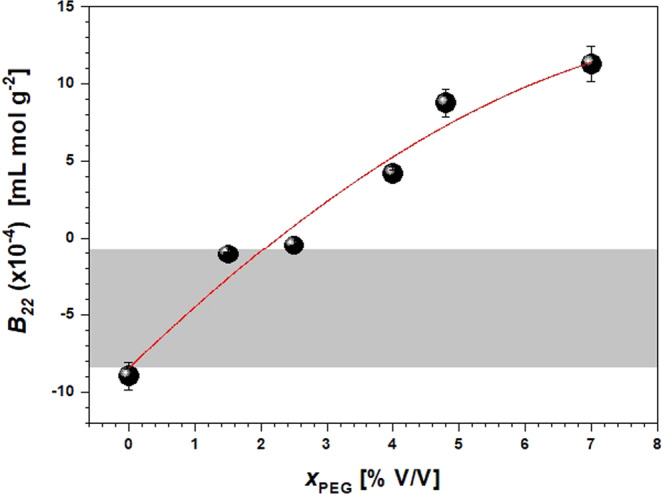
The second virial coefficient *B*_22_ as a function of PEG400 volume concentration (*X*_PEG_) at a constant amount of 0.48 M Na_2_SO_4_ in HEPES 0.1 M buffer at pH 7.4. The crystallization slot according to ref. ^15^ is shown in light grey. The solid line is a guide for the eyes.

**Figure 4:**
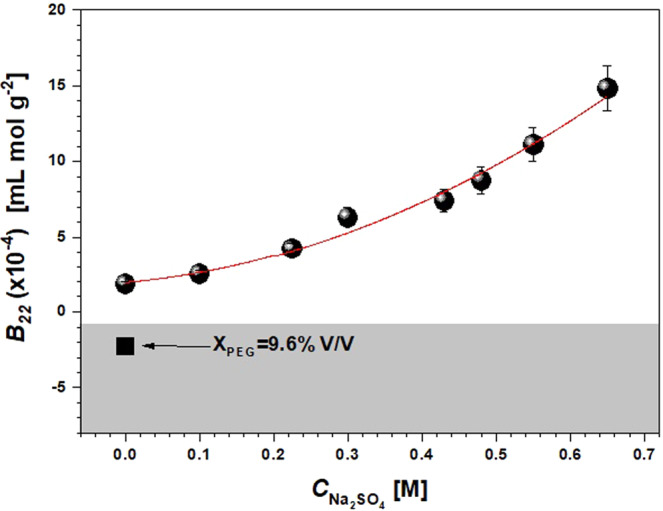
The second virial coefficient *B*_22_ as a function of Na_2_SO_4_ molar concentration (${C}_{N{a}_{2}S{O}_{4}}$) for constant PEG400 volume concentration *x*_PEG_ = 4.8% V/V in HEPES 0.1 M, pH 7.4. The solid square symbol indicate *B*_22_ for a PEG400 concentration of 9.6% V/V without NaCl. The crystallization slot according to ref. ^15^ is shown in light grey. The solid line is a guide for the eyes.

It is well known that the addition of salts can cause an electrostatic double layer around the protein surface charges, which involves a shielding and the reduction of repulsive interactions among macromolecules. Furthermore, the salts ions compete with the protein for water molecules and dehydrate the protein (salting out effect^37^), thus inducing aggregation by mainly electrostatic and hydrophobic interactions. On the other side, polymers such as PEG bring the protein molecules together due to preferential interactions and osmotic potential^38^. Accordingly, the addition of PEG400 or Na_2_SO_4_ to Anti-CD20 solutions is expected to increase the aggregation aptitude of the protein and facilitate crystallization, as experimentally observed, e.g., in batch crystallization tests of Table 1. However, as displayed in Figs. 3 and 4, for increasing amounts of PEG or Na_2_SO_4_ when both components are present in solution, the *B*_22_ moves towards larger positive values, theoretically indicative of an increase in repulsive interactions. Furthermore, effective Anti-CD20 crystallization conditions seems to lay outside the crystallization slot defined by George and Wilson^15^.

Both Figs. 3 and 4 show that the combined effect of PEG400 and Na_2_SO_4_ has a noteworthy consequence on the *B*_22_ value. For instance, for the solution containing 0.48 M Na_2_SO_4_ in absence of PEG, a *B*_22_ value of −8.9 × 10^−4^ mL mol g^−2^ is obtained (Fig. 3) that is slightly below the crystallization slot. However, when adding PEG400 to the solution while keeping salt concentration constant, *B*_*22*_ steeply increases through the crystallization window towards positive values already at 3% V/V of PEG.

The *B*_22_ value for the solution containing 4.8% V/V PEG400 in absence of Na_2_SO_4_ is found to be slightly positive (*B*_22_ = 1.8 × 10^−4^ mL mol g^−2^), becoming negative (*B*_22_ = −2.26 × 10^−4^ mL mol g^−2^) when increasing PEG400 up to *x*_PEG_ = 9.6% V/V without Na_2_SO_4_ (Fig. 4). This is in agreement with results from Ahamed *et al*.^39^, who observed the reduction in solubility for a mAb for increasing amounts of PEG400. Nevertheless, when salt is added to the solution (${C}_{N{a}_{2}S{O}_{4}}\ne 0$), the virial coefficient rises steeply with the salt concentration. These observations clearly indicate that a different effect on the measured value of *B*_22_ takes place when PEG400 and Na_2_SO_4_ are combined together, compared to the cases when they are used separately.

**Figure 5:**
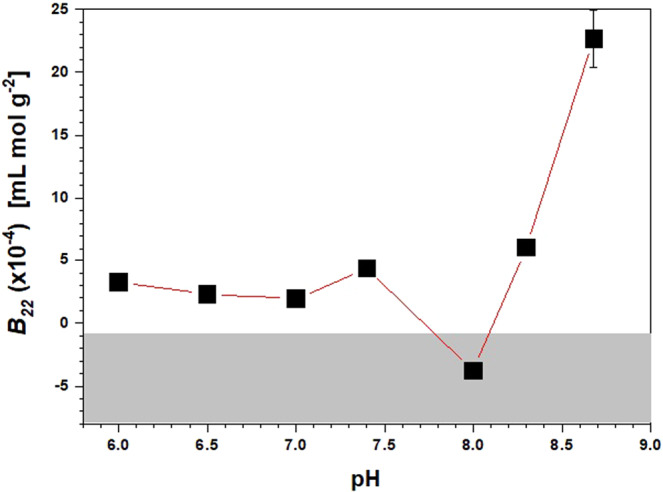
pH dependence of *B*_22_ for Anti-CD20 solutions with 4.8% V/V PEG400, 0.43 M Na_2_SO_4_, and HEPES 0.1 M. The crystallization slot according to ref. ^15^ is shown in light grey. The solid line is a guide for the eyes.

Figure 5 shows the effect of pH on *B*_22_ for solutions containing PEG400 and Na_2_SO_4_. At pH ≤ 7.6, the *B*_22_ remains roughly unchanged (2.28 < *B*_22_ < 4.35 × 10^−4^ mL mol g^−2^). These positive values are indicative of repulsive protein–protein interactions. At a pH of 8.0, the *B*_22_ becomes negative (−3.78 × 10^−4^ mL mol g^−2^) and then sharply increases approaching pH = 8.8, which is the theoretical isoelectric point of Anti-CD20 (p*I* = 8.8)^40^.

It is expected that protein-protein interactions at a pH substantially below or above the p*I* are repulsive, while at a pH near the p*I* they become increasingly attractive due to the overall neutral charge of the protein. However, the actual p*I* of biomolecules might change with ionic environment, and generally decreases at different extents with increasing ionic strength, depending on the nature of the counter-ion^41,42^. It is speculated that in the presence of 0.43 M Na_2_SO_4_, PEG400 4.8% V/V and 0.1 M HEPES, the effective p*I* of Anti-CD20 is around 8.0. Therefore, for pH < 8.0, the slightly positive *B*_22_ values are most likely due to partially screening of the net positive electrostatic charges of protein molecules, while at pH = 8.0, where the net charge becomes null, the net resultant interaction is attractive. Above pH 8.0, the *B*_22_ values become strongly positive, indicating an ineffective screening of the net negative charge of molecules that results in repulsive interactions.

**Figure 6:**
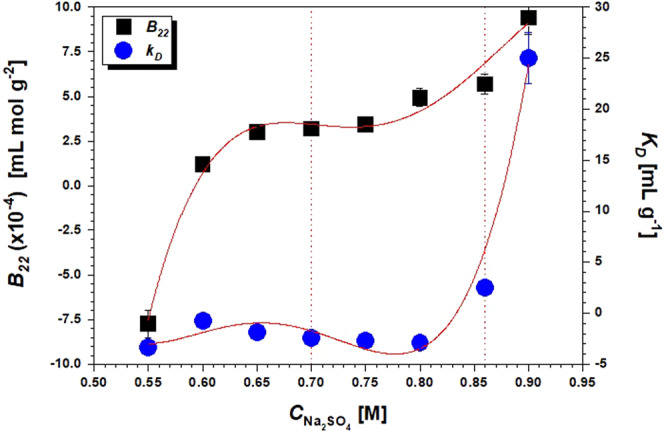
*B*_22_ and *k*_d_ as a function of Na_2_SO_4_ concentration for *x*_PEG_ = 9.6% V/V and pH 7.4. The experimental crystallization window boundaries (see Table 1) are highlighted by the dotted vertical lines. The solid lines are guides for the eyes.

Since solution composition strongly affects protein stability and aggregate formation, the Na_2_SO_4_ concentration dependence of *B*_22_ was also investigated at higher PEG and salt concentrations, thus reaching compositions close to that used for crystallization experiments of Table 1. Correlation between *B*_22_ and Na_2_SO_4_ concentration is shown in Fig. 6, confirming an increasing trend similar to that observed for lower concentrations of Fig. 4 also in this case.

In Fig. 6 it can be seen that the *B*_22_ values are negative (attractive interactions) for Na_2_SO_4_ concentration ranging from 0.55 to 0.6 M, corresponding to conditions in Table 1 that give clear solutions, while they become positive (repulsive interactions) when the salt concentration is further increased up to 0.9 M. Within the crystallization window, i.e. Na_2_SO_4_ ranging from 0.7 to 0.86 M, the *B*_22_ values range from 3.2 × 10^−4^ to 5.7 × 10^−4^ mL mol g^−2^. This would be theoretically indicative of the absence of attractive colloidal interactions, in opposition to the results of the crystallization tests.

DLS measurements were then used to determine the interaction parameter *k*_d_. Generally, *k*_d_ and *B*_22_ display a qualitatively similar trend^43^. However, looking at the behaviour of the interaction parameter *k*_d_ in the crystallization range of Fig. 6, negative values are found for *x*_PEG_ = 9.6% V/V and sodium sulfate 0.55–0.80 M, with slight variations within this range. Generally, a negative value of *k*_d_ implies the existence of attractive interactions between protein molecules which slow down their diffusion in solution^44^. Since under the same conditions *B*_22_ is negative only for Na_2_SO_4_ < 0.6 M and then increases up to 1.23–4.94 × 10^−4^ mL mol g^−2^ in the salt concentration range 0.6–0.8 M, this indicates that underlying attractive interactions do not have a thermodynamic origin of colloidal nature. We can instead conclude that these interactions result from the hydrodynamic effects included in the second term of Eq. 4^45^, most likely due to the increase of solution viscosity (from 1.949 cP in the PEG400 9.6% V/V solution without salt to 2.546 cP for PEG400 9.6% V/V and Na_2_SO_4_ 0.9 M). Nevertheless, as for *B*_22_, the increasing trend of *k*_d_ with the concentration of Na_2_SO_4_ does not comply with the outcome of crystallization tests. In fact, the increase of Na_2_SO_4_ concentration at values larger than 0.8 M gives rise to a theoretically significant increase of the strength in protein-protein repulsions, according to measured *B*_22_, values that are exacerbated near the LLPS region, while crystals are obtained in such conditions.

From the observed trends of *B*_22_ and *k*_d_, we can conclude that neither PEG400 nor Na_2_SO_4_ act as conventional precipitants for Anti-CD20 when used together since, on average, Anti-CD20 monomers apparently repel one another. Nevertheless, this evidently contradicts the experimental outcomes of crystallization tests. The *B*_22_ values are negative (attractive interactions) in formulations that remain clears for months, whereas positive values, corresponding theoretically to net repulsive interactions, have been found for the best-performing crystallizing or either in precipitating conditions.

Considering the possible variations in their structure, morphology, size, surface charge and chemistry, proteins definitely represent one of the most complex colloidal systems. Several mechanisms could lead to molecular aggregation in solution and it is not excluded that more than one is active in certain conditions. Therefore, it is not surprising that *B*_22_ does not always reflect the aggregation propensities of proteins in a given formulation. Several papers can be found in literature reporting discrepancies between *B*_22_ and the susceptibility to aggregation of structurally complex macromolecules, like monoclonal antibodies^43,46^. Crystallizing conditions with positive *B*_22_ values were found for a mAb molecule with PEG400 by Rakel *et al*.^35^. This behaviour has been ascribed to the ambivalent character of PEG400 as precipitant and stabilizer^35,47^. Kress *et al*.^48^ observed the decrease in *B*_22_ of an immunoglobulin G (IgG) when increasing PEG2000, sodium citrate and NaCl solute concentration, if used separately. However, the mutual effect of PEG and salts resulted in a shift of *B*_22_ from negative to almost neutral values when increasing the NaCl amount if used together^32^. This behaviour has been explained by a solubilizing effect of IgG in PEG2000 induced by NaCl until a maximum salt concentration. Exceeding this NaCl value, a solid-liquid equilibrium, due to protein precipitation, was observed. Herhut *et al*.^49^ measured *B*_22_ for an IgG and D-xylose ketol-isomerase (172.3 kDa) with ammonium sulphate and PEG12000 and PEG2000, respectively. They observed the initial decrease in *B*_22_ at low PEG concentration, passing through a minimum and then increasing for higher PEG concentrations. This behaviour has been explained by considering that polymer-induced interactions are not solely attractive over the whole polymer concentration range. Normally, polymer chains behave as coil-inducing attractive interactions between protein molecules at low-polymer concentration. However, at high polymer concentration, flexible coils elongate and enter space between the proteins, so that repulsive interaction are induced^50,51^. This explanation has been also confirmed by molecular dynamic simulations at different polymer concentration by Cao *et al*.^52^.

It has also been reported that conformational stability plays an important role in aggregation propensity, with partially unfolded conformational intermediates being responsible of aggregate formation^9^. In this case, the *B*_22_ value is unlikely to correlate with long-term aggregation because the structurally perturbed state susceptible to aggregation could be present in a small fraction of molecules compared to the native species^46^. Aiming to shed light on this aspect, protein structure in solution has been investigated in the present work.

### Protein structure in the solution

On the bases of the results above, measurements of melting temperature (*T*_*m*_) for several solution compositions, including *x*_PEG_ = 9.6% V/V and Na_2_SO_4_ at various concentrations, and Circular Dichroism (CD) in selected formulations, were performed aiming to investigate conformational stability of Anti-CD20. Results show slight variations of the melting points: *T*_*m*_ = 67.0, 67.5, 68.0, and 67.5 ± 0.5 °C for 0.55, 0.65, 0.75, and 0.85 M Na_2_SO_4_, respectively, confirming that samples, on average, share similar water activity according to the Wyman-Tanford equation^53^.

**Figure 7:**
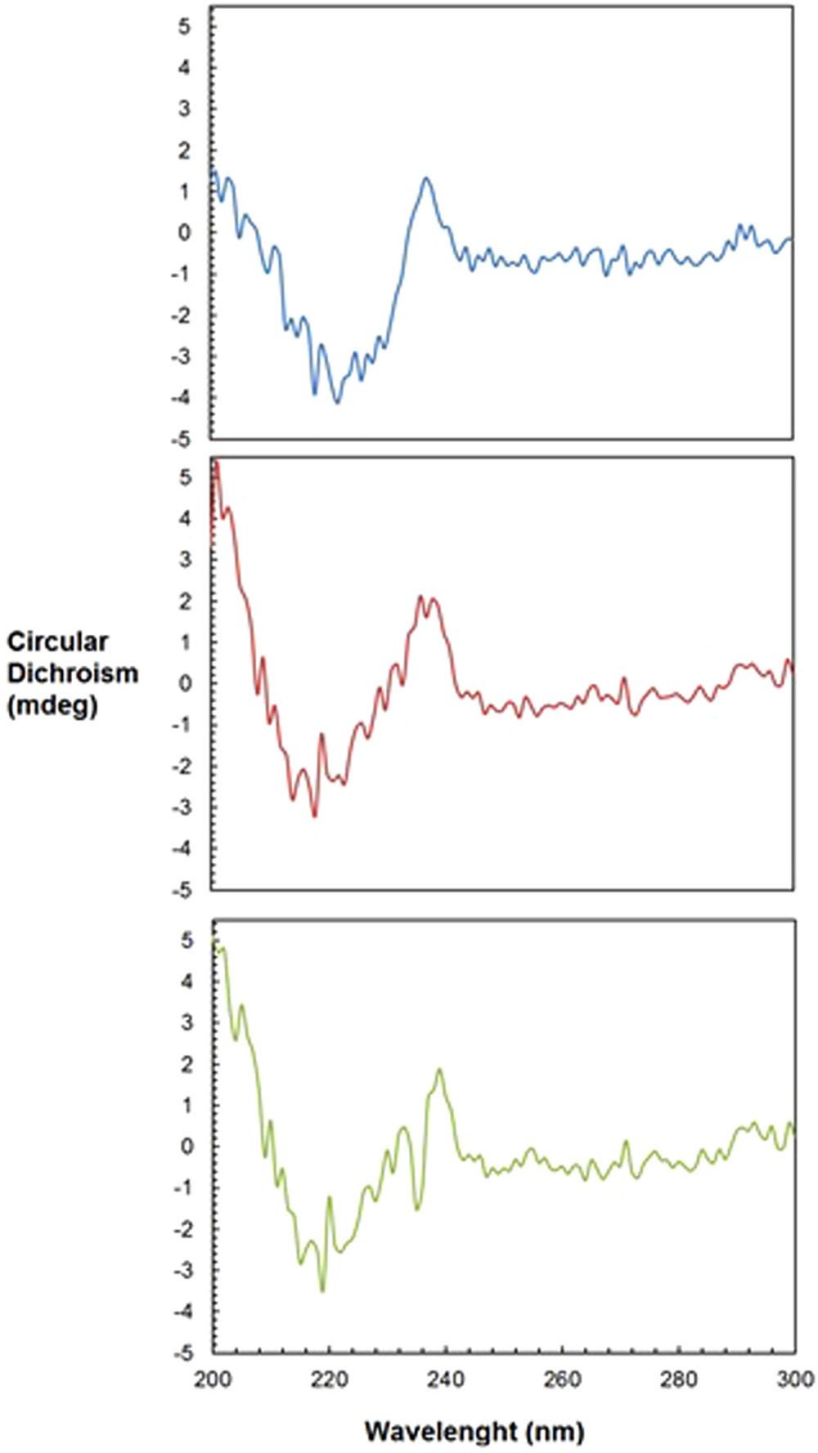
Far-UV CD spectra of Anti-CD20 under initial conditions (blue spectra, mAbs concentration 9.95 mg mL^−1^ in stock buffer), after buffer exchange with HEPES 0.1 M pH 7.4 (red spectra, mAb concentration 100 mg/mL in crystallization buffer) and after salt mixing (green spectra, mAb concentration 60 mg/mL in crystallization buffer and with 0.9 M Na_2_SO_4_ and 9.6% V/V PEG400).

Circular Dichroism spectra of protein solutions give information on the secondary structure, folding properties and conformational variation of the proteins^54^. The general shape of the spectrum of the provided Anti-CD20 solution (Fig. 7, initial conditions) corresponds to the expected spectra for Anti-CD20, as it shows the typical shape of a monoclonal antibody with a minimum around 220 nm and a maximum around 200 nm, suggesting a secondary structure dominated by β-sheet motif^54^. Moreover, these spectra correspond to the CD-spectra of Anti-CD20 from literature^55^.

These spectra show that the protein is well folded in every buffer, i.e. the 3D-structure of Anti-CD20 is preserved under all conditions tested. However, the maximum ellipticity (around 200 nm) is much higher after the buffer exchange (red and green spectra in Fig. 7) than in the initial buffer (blue spectrum), while ellipticity at other wavelengths is similar in every buffer. This suggest that the β-sheet motifs of the mAbs are favoured and stabilized in the new HEPES buffer, in comparison to the initial buffer (0.025 M sodium citrate at pH 6.5 and 0.154 M sodium chloride).

There is no significant difference in the far-UV CD spectra of Anti-CD20 before (Fig. 7, red spectrum) and after the addition of Na_2_SO_4_ and PEG400 as precipitating agents (Fig. 7, green spectrum), although a small shift can be observed, showing slight changes in the 3D-structure. The main difference between these spectra is around 230 nm, but the peak is quite narrow and this wavelength does not correspond to any typical feature of protein structure. Therefore, the difference could be due to Na_2_SO_4_ or PEG400 absorbance or might be due to some noise. It should be noted, though, that the crystallization solution had to be diluted in order to enable CD measurement. Since the spectra before and after the concentrated crystallization conditions are the same, it can be concluded that the concentrated crystallization conditions do not alter the secondary structure of the protein.

The Anti-CD20 secondary structure is preserved after centrifugation and addition of the precipitating agents, as the typical β-sheet motif appears clearly in the spectrum (Fig. 7, green spectrum). Moreover, this secondary structure seems to be favoured in the crystallization buffer, meaning that the 0.1 M HEPES at pH 7.4 buffer promotes Anti-CD20 crystallization by making the secondary structure less flexible. However, no clear effect of Na_2_SO_4_ or PEG400 on the Anti-CD20 secondary structure is visible.

### The role of LLPS in Anti-CD20 crystallization

A complementary scenario can be envisioned when considering LLPS phenomena observed concurrently or just prior of Anti-CD20 crystallization.

For simple protein/salts or protein/PEG systems, it has been often reported that the *B*_22_ value can be used as a predictor of LLPS formation, since weak protein-protein interactions, in general hydrophobic in nature or even limited to specific protein chain sequences, are at the origin of the phase separation^22^. However, our results seem to suggest that the LLPS (observed just prior or at the point of protein crystallization) proceeds without attractive interactions, since *B*_22_ is negative when a mono-phase system is observed while it turns positive as approaching the liquid phase separation and crystals formation.

It could be argued that, in our complex mixture that includes several components, the *B*_22_ values fail to predict the experimentally observed crystallization since it may be rather related to the polymer-salt phase separation that can occur without protein. The liquid-liquid separation of PEG/salt aqueous solutions is a fundamentally different phenomenon from PEG/protein or salt/protein LLPS, which originate by a combination of hydrophobic and salting-out effects, whose origin has been attributed to the different conformations of the PEG polymer chain in aqueous solution and their respective hydration^24^. Actually, the PEG400/Na_2_SO_4_/H_2_O system is known to generate aqueous two-phase systems^36,56,57^. Figure 2 displays the experimental binodal curve together with liquid-liquid equilibrium tie-lines data for PEG400 + Na_2_SO_4_ + H_2_O system at 293.15 K (the temperature used in this work for crystallization experiments and SLS/DLS measurements)^36^.

Concerning the specific system studied in this work, for a protein concentration of 20 mg mL^−1^ and a PEG400 content of 9.6% V/V (9.4 wt.%), phase separation is observed for salt concentrations ≥0.7 M (≥8.6 wt.%) (crystallization points of Table 1 and dark circles in Fig. 2), that is quite far from both binodal curve. However, the presence of the protein can reduce the amount of salt needed for observing LLPS in the multicomponent PEG/salt/protein/water systems^24^. Consequently, it cannot be excluded that the effective binodal curve for PEG/salt LLPS is shifted down to conditions corresponding to the observed crystallization points, in a region of poor solvent conditions, thus giving a decisive contribute to the aggregation/crystallization behaviour of Anti-CD20.

**Figure 8:**
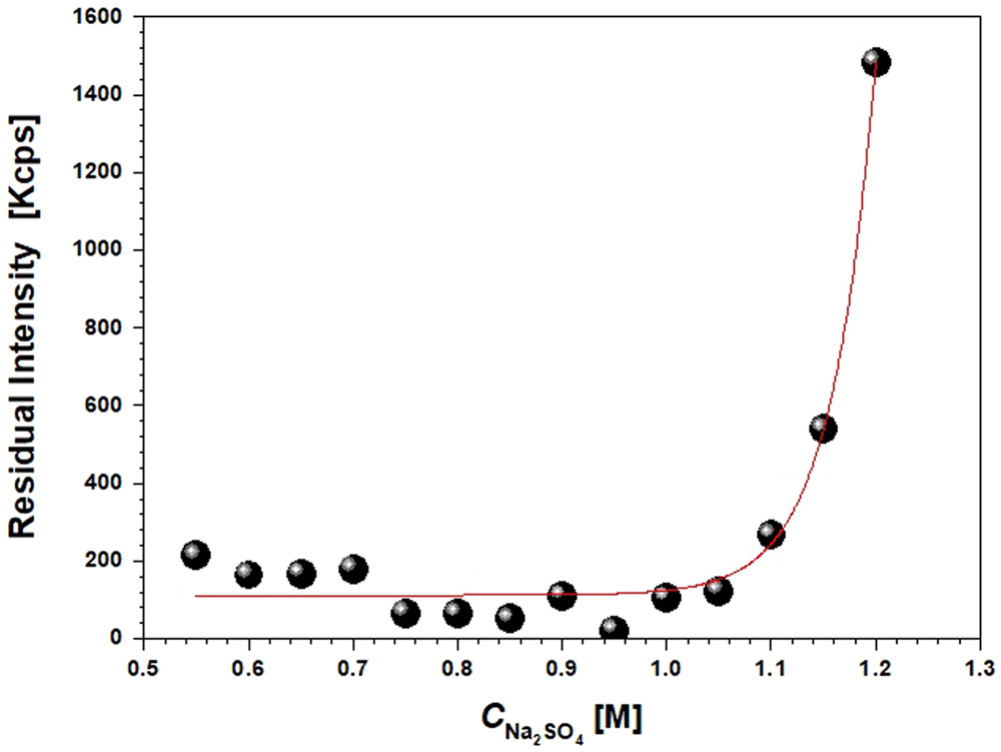
Residual Intensity measured by DLS at a fixed scattering angle of 173° for a solution containing PEG400 9.6% V/V in 0.1 M HEPES at pH 7.4 and increasing amount of Na_2_SO_4_, without protein. The solid line is a guide for the eyes.

Figure 8 shows the change in turbidity (Residual Intensity) for a solution containing PEG400 9.6% V/V in 0.1 M HEPES at pH 7.4 and increasing amount of Na_2_SO_4_. It is observed that, in absence of the Anti-CD20, a sharp increase in solution turbidity is observed for salt concentration >1.1 M (>13.5 wt.%), that is very close to the binodal curve reported in Fig. 2 for the PEG400 + Na_2_SO_4_ + H_2_O system. Since in the presence of the protein turbidity due to liquid-liquid phase separation is observed for salt concentration larger than 0.65 M for the same amount of PEG, this proves the shift of LLPS region towards lower values of PEG and/or salt components when Anti-CD20 is present in the solution.

It is worth noting that *B*_22_ measurements reported in Figs. 3 and 4 have been made in a region of Fig. 2 where LLPS (and crystallization) are not observed (triangles in the left-bottom side of the figure). This means that PEG/salt LLPS is not responsible for the contradiction between *B*_22_ measurements and the outcome of crystallization trials, but rather the intrinsic complexity of the system makes ineffective the measurement of the osmotic coefficient as predictor parameter for crystallization conditions.

According to our results, it can be proposed that PEG/salt LLPS causes the preferential partitioning of proteins in a specific salt- or PEG-rich phase. In general, the affinity of the protein for the polymeric component, due to hydrophobic interactions, favours the protein concentration in the PEG-rich phase^39,58^. This is particularly true for PEG400, which is a low molecular weight polymer and, therefore, its size exclusion effects on proteins are negligible while the dominant factor in the separation is the salting out effect, produced by the sulphate, which causes the protein to migrate to the PEG-rich phase. This effect has been confirmed by measurements on Anti-CD20 partitioning, showing that more than 90% of the solution protein migrates in the PEG-rich phase in the protein-saturated Anti-CD20/PEG400/Na_2_SO_4_/HEPES/H_2_O mixture displaying phase separation.

**Figure 9:**
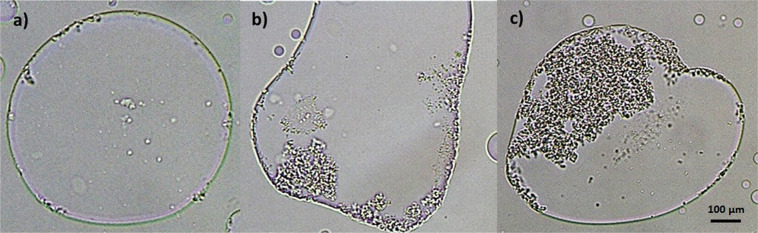
Protein crystals nucleating near the boundary of the droplets (**a**) and crystals forming within the bulk of the droplets (**b**,**c**).

In such conditions, during LLPS, the Anti-CD20 concentrates in droplets that have a density higher than the average density of the whole system thus generating a much higher supersaturation. When the protein concentration exceeds a certain limit, precipitation at the interface is observed and the precipitated protein is in equilibrium with the protein solubilised in each phase^59^. For particularly high Anti-CD20 (>30 mg mL^−1^ or PEG/salt concentrations (i.e. PEG400 9.4 wt.% and Na_2_SO_4_ ≥ 11.1 wt.%), these droplets look not to be as liquid, having instead the appearance of gel beads. Protein crystals initiate to nucleate and grow near the boundary of the most protein-concentrated droplets (Fig. 9), suggesting that the droplet interface participates in the nucleation mechanism^60^. Subsequently, crystals appear within the bulk of the droplets, which spread and coalesce. Many droplets disappear as crystal growth proceeds, suggesting that a continuous exchange exists between the droplets and the surrounding medium.

Experimental observations clearly indicate the important role of LLPS and of the established liquid-liquid interface in inducing Anti-CD20 nucleation, even in absence of underlying net attractive interaction between proteins. It is likely that only protein molecules distributed at the droplets interface experience local short-range attractive interactions, due to the specific environment and/or conformational changes, not influencing the mean *B*_22_ value.

On the basis of our results, it could be hypothesized that the occurrence of LLPS in crystallizing/precipitating conditions results in a partially unfolded protein conformation or in a suitable variation of the secondary/tertiary protein structure, as already reported in other cases^27^, which can be the species prone to aggregation through a mechanism driven by conformational change rather than colloidal interactions, and which initiate the crystallization process. The conformationally perturbed protein can be a minor species, which does not alter significantly the predominant native protein population and only slightly contributes to the net protein–protein interaction value measured by *B*_22_. Indeed, SLS is an integrative technique, which provides only averaged information. If only specific short-range orientations or aggregation-prone structurally perturbed states can lead to attractive interactions, the remaining repulsive orientations or species dominate in the light scattering results.

It is important to note that the measurements of intermolecular interactions were performed under low protein concentration conditions (≤5 mg mL^−1^), while crystallization trials are generally conducted at higher concentrations (≥20 mg mL^−1^). Both *B*_22_ and *k*_d_ determined in dilute conditions are considered generally predictive of high-concentration behaviour. Nonetheless, high concentration behaviour can differ from that observed at dilute conditions, since “crowding” effects, i.e. the increased chance of molecular interactions, deviation from thermodynamic ideality and higher-order interactions (e.g., *B*_23_, *B*_222_) can significantly alter the net inter-protein interactions^55,61^. The contribution of these effects to aggregation propensity of structurally complex molecules such as mAbs, cannot be ruled out.

## Conclusions

This work reports about the study of Anti-CD20 (a full-length monoclonal antibody) crystallization in a PEG400/Na_2_SO_4_/H_2_O system occurring near LLPS conditions. The DLS/SLS studies performed to verify the correlation between the second virial coefficient and molecular diffusivity with the protein aggregation propensity in selected solution formulations indicates that *B*_22_ remains strongly positive (a sign of repulsive protein–protein interactions) for all conditions that have been instead ascertained to be effective for protein crystallization or precipitation. For all the crystallizing or precipitating conditions, the occurrence of LLPS phenomena was observed concomitantly or near the nucleation stage. Even in this case, the second virial coefficient does not correlate with the LLPS, which mainly proceeds without attractive protein interactions, and that may be rather related to the polymer-salt phase separation that can occur without protein. The possible interplay between the salt/PEG and protein/PEG phase separation, which can affect the solvent quality, can have a role on the polymer mediated forces and finally on Anti-CD20 aggregation. On the basis of these measurements and the experimental observation of LLPS near the nucleation zone, it is proposed that a minor population of protein molecules can experience valuable protein-protein attractive interactions, induced by local environmental factor, slightly affecting the averaged *B*_22_ parameter.
